# Continuous Patterning of Silver Nanowire-Polyvinylpyrrolidone Composite Transparent Conductive Film by a Roll-to-Roll Selective Calendering Process

**DOI:** 10.3390/nano13010032

**Published:** 2022-12-21

**Authors:** Hakyung Jeong, Jae Hak Lee, Jun-Yeob Song, Faizan Ghani, Dongjin Lee

**Affiliations:** 1 Department of Ultra-Precision Machines and Systems, Korea Institute of Machinery and Materials (KIMM), Daejeon 34103, Republic of Korea; 2 Department of Mechanical Design and Production Engineering, Konkuk University, Seoul 05029, Republic of Korea; 3 Department of Mechanical and Aerospace Engineering, Konkuk University, Seoul 05029, Republic of Korea

**Keywords:** roll-to-roll, continuous patterning, selective calendering, silver nanowire-polyvinylpyrrolidone composite, flexible electronic device

## Abstract

The roll-to-roll (R2R) continuous patterning of silver nanowire-polyvinylpyrrolidone (Ag NW-PVP) composite transparent conductive film (cTCF) is demonstrated in this work by means of slot-die coating followed by selective calendering. The Ag NWs were synthesized by the polyol method, and adequately washed to leave an appropriate amount of PVP to act as a capping agent and dispersant. The as-coated Ag NW-PVP composite film had low electronic conductivity due to the lack of percolation path, which was greatly improved by the calendering process. Moreover, the dispersion of Ag NWs was analyzed with addition of PVP in terms of density and molecular weight. The excellent dispersion led to uniform distribution of Ag NWs in a cTCF. The continuous patterning was conducted using an embossed pattern roll to perform selective calendering. To evaluate the capability of the calendering process, various line widths and spacing patterns were investigated. The minimum pattern dimensions achievable were determined to be a line width of 0.1 mm and a line spacing of 1 mm. Finally, continuous patterning using selective calendering was applied to the fabrication of a flexible heater and a resistive touch sensing panel as flexible electronic devices to demonstrate its versatility.

## 1. Introduction

Recently, extensive studies have been carried out on devices with flexible, stretchable, and wearable characteristics in the electronics industry. Flexible electronic devices such as transistors, electronic paper, memory, organic light-emitting diodes, sensors, solar cells, and batteries [1,2,3] have been reported using micro/nano material- and structural-based technologies. The flexibility must be approached from the complex viewpoint of materials and manufacturing processes, including miniaturization, lightweight, efficiency, and yield. While the thin film structures of metals and composites has conventionally been manufactured using electrochemical deposition [4,5,6] and ion-beam sputtering [7] as the batch type, the roll-to-roll (R2R) process has endowed electronic devices with more flexibility and mass-producibility. Silver nanowires (Ag NWs) are still receiving great attention as flexible electronic materials, even though many reports with a variety of manufacturing technologies have been published over a long period of time. Ag NWs are formed with a diameter of several nanometers, which have excellent electronic, optical, and mechanically stable properties [8,9,10]. In addition, they have a very low percolation threshold when they form random networks. The production cost of Ag NW thin films had been more expensive than indium tin oxide (ITO) films. However, it gradually became cheaper because only a small amount of solution is necessary for high-quality thin films and a variety of manufacturing technologies can be adopted. Regardless of the various advantages, several problems in Ag NWs exist: (1) aggregation, (2) oxidation, (3) surface roughness, (4) adhesion onto the substrate, and (5) limitations in the patterning scheme. In this work, R2R slot-die coating and a calendering process were adopted to manufacture thin films of silver nanowire composite where some of these problems were addressed.

A dispersant is commonly used for nanomaterials since aggregation is one of their intrinsic characteristics. Polymeric material has been generally used as a dispersant [11,12]. Various polymeric dispersants such as polyurethane (PU), polymethyl methacrylate (PMMA) [13], polyaniline (PANI), polyacrylic acid (PAA), and polyvinyl pyrrolidone (PVP) [14] have been used depending on functional groups on the surface of the nanomaterials. Polymeric dispersants have different interactions with regard to the nanostructure; thus, understanding the chemical reaction in the synthetic process and determining suitable polymer matrices are important to choose the appropriate dispersant. Ag NW composites with dispersants have helped to improve the dispersion and construct an effective conductive percolation network at the same time. PVP is one of the amphiphilic polymers that includes a pyrrolidone hydrophilic moiety and an alkyl hydrophobic moiety. It has good solubility in many organic solvents and water because hydrogen bonds are generated among the carbonyl groups [15]. Moreover, PVP acts as a capping agent in the synthesis of Ag NWs. Therefore, we have used a Ag NW-PVP composite to fabricate transparent conductive films in this work. The Ag NW composite is an advanced material that functionalizes various properties [16,17] for various applications such as in chemical/physical sensors [18,19], supercapacitors [20,21], photovoltaics [22], and energy storage [23].

Our previous work demonstrated an R2R calendering process for pure Ag NW thin films [9]. The general purpose of calendering is to improve the performance of multilayered electronic devices by making smooth surfaces and interfaces. It is a simple and fast method where various manufacturing processes are easily grafted. Calendered Ag NW thin films showed an increased electrical conductivity of more than 100 times, and a decreased surface roughness of one-half. However, one of most critical issues with Ag NW thin films in practical applications is the patterning process. The patterns of Ag NW thin films were achieved by various methods such as photolithography [24,25], templated assembly [26,27,28,29], laser ablation [30], surface wettability control [31,32,33], chemical etching [34,35,36], electrospray [37], thermocompression [38], inkjet printing [39], and gravure printing [40]. However, some of these suffer from the disadvantages of complexity, low quality, chemical damage, and poor feasibility due to a long process time. Moreover, the weak bonding forces among Ag NWs as well as between the Ag NW thin film and substrate are obstacles for high electrical performance and mechanical stability. To strengthen adhesion to the substrate, a variety of methods have been studied previously, e.g., encapsulation [41,42], surface functionalization [43], photonic sintering [44], composite with functional material [45,46,47], and compression [48]. However, these methods require an additional equipment set-up and cannot be integrated into the existing manufacturing process. The best outcome was that strong adhesive Ag NW thin films were manufactured with patterning capability. As a further step from our previous report on calendering of Ag NW films, therefore, we employed an embossed roll to perform the calendering process, which is called selective calendering. It was integrated into R2R slot-die coating machine where a Ag NW-PVP composite film was formed.

The present work deals with the patterning of Ag NW-PVP composite transparent conductive films (cTCF) by a selective calendering process. PVPs with various weight percents and molecular weights were utilized to evaluate effect of calendering of Ag NW-PVP cTCFs. The weight percent influences the sheet resistance directly because the remaining PVP acts by interrupting the percolation path. In addition, the molecular weight is associated with the dispersion that determines the distribution of Ag NWs in cTCFs. The Ag NW-PVP composite improved the dispersion of Ag NWs and resistance to oxidation by capped PVP, which resulted from a simplified washing scheme after synthesis. Furthermore, the electrical conductivity was extremely increased by the calendering process. For this reason, the patterning is enabled by selective calendering using an embossed roll. The suggested patterning process is integrated into an existing R2R slot-die coating machine, which allows rapid production of various conductive electrodes patterns.

## 2. Materials and Methods

### 2.1. Synthesis of PVP-Capped Ag NWs

The polyol method was adopted in this work for the mass-production of Ag NWs and ease of composite formation. The detailed procedure was published previously in ref. [49]. All chemical reagents including ethylene glycol (EG) as a solvent, polyvinylpyrrolidone (PVP, M_w_~40,000) as a capping agent, copper chloride (CuCl_2_) as a reducing agent, and silver nitrate (AgNO_3_) as a precursor were purchased from Sigma-Aldrich. First, 50 mL of EG in a round-bottom flask was preheated in an oil bath at 151.5 °C. After enough heating until thermal stabilization, CuCl_2_ (400 μL, 4 mM) and PVP (15 mL, 0.147 M) solutions in EG were added into the flask with a time interval of 15 min. Finally, a AgNO_3_ (15 mL, 0.094 M) solution in EG was prepared by ultra-sonication for 5 min and was injected at a rate of 0.5 mL/min by a syringe pump. The reaction proceeded for 3 h and the color of the mixed solution was changed to silver gray. Then, the flask was removed from the oil bath and cooled down to ambient temperature. The as-synthesized Ag NW solution was mixed with acetone in a volume ratio of 1:10. After gentle mixing by hand and 10 min incubation, the supernatant, except for the Ag NW precipitate, was discarded. To compare the effect of washing, additional washing with ethanol was also considered. In this case, the precipitated Ag NW was re-dispersed in ethanol and centrifuged at 5000 rpm for 20 min and the supernatant was discarded. In the absence and presence of additional washing, finally, the Ag NW precipitate was dispersed in isopropyl alcohol (IPA) with a concentration of 1.5 wt%. The PVPs possessing various molecular weights were dissolved in IPA with concentrations of 0, 0.25, 0.5, 0.75, and 1 wt% to investigate the effect on the selective calendering process. The prepared Ag NW-PVP composite solution was used as the coating solution for the R2R slot-die process.

### 2.2. Fabrication of Patterned Ag NW-PVP Composite TCF

The effect of the calendering process on Ag NW films has been studied in ref. [9]. It was found that the random network of mechanically pressed Ag NWs shows much lower sheet resistance than the unpressed one. Instead of a flat roll, we employed an embossed roll to perform the calendering process, which is called selective calendering. The slot-die coating of the Ag NW-PVP composite was performed in an in-house coating machine. The flow rate of coating solution was controlled using a syringe pump (LEGATO^®^ 100, KD Scientific Inc., Holliston, MA, USA) connected to an inlet for the slot-die coater. The dryer was composed of far infrared (FIR) heating units with convection. The roll behind the dryer was used with the aid of a nip roll as the calendering roll. Depending on the purpose, a flat roll or an embossed roll was employed for full area calendering or selective calendering, respectively. The operating parameters were web speed, tension force, and nip roll pressure. The default operating conditions were 1 m/min web speed, 1 kgf of tension, and 5 bar of nip roll pressure. In addition, a flow rate of 2 mL/min and a drying temperature of 90 °C were used. Unless noted, the default manufacturing conditions were used. To investigate the effect of washing and of the PVP in coating solution, the flat roll was used as a calendering roll. For the patterning purpose, the embossed roll (IL SHIN TECH Co., Ltd., Bucheon-si, Gyeonggi-do, Republic of Korea) was exploited. The depth of the embossed roll was fixed at 100 μm.

### 2.3. Measurement and Characterization

The morphology and surface roughness of the Ag NW-PVP cTCF were measured with field-emission scanning electron microscopy (FE-SEM, S-4800, Hitachi, Japan) and atomic force microscopy (AFM, XE-100, Park Systems, Suwon-si, Gyeonggi-do, Republic of Korea), respectively. For measuring sheet resistance, the sample was cut into square shapes of 16 cm^2^ area, and copper tapes were attached on both sides. The resistance was measured by a source meter (2611A SYSTEM SourceMeter^®^, KEITHLEY, Solon, OH, USA). Transmittance measurements were carried out with UV–Visible spectroscopy (Optizen 2120UV, MECASYS, Daejeon, Republic of Korea). In order to carry out the analysis of the chemical elements, energy dispersive spectrometry (EDS) was used which was connected to a FE-SEM (JSM-7610F Plus, JEOL, Akishima, Tokyo, Japan).

### 2.4. Applications of the Patterned Ag NW-PVP Composite TCF

To demonstrate the usefulness of the Ag NW-PVP cTCF, two kinds of functional devices were fabricated. Firstly, a flexible transparent heater used zig-zag patterns of cTCF was fabricated. After formation of zig-zag patterns by selective calendering, copper tape was attached to the square-shaped pads at the beginning and end of the zig-zag line. To reduce the contact resistance, silver paste was applied to the edge of copper tape and dried. An operating voltage of 5 V was applied to the copper tape. Joule heating characteristics were measured by a far infrared camera (SC660, Teledyne FLIR LLC, Wilsonville, OR, USA). Secondly, a resistive touch sensing panel was fabricated as has already been reported in ref. [9]. The upper and bottom electrodes were both Ag NW-PVP cTCFs fabricated in this study, although the calendered part was different.

## 3. Results and Discussions

### 3.1. Ag NW-PVP Composites

**Figure 1:**
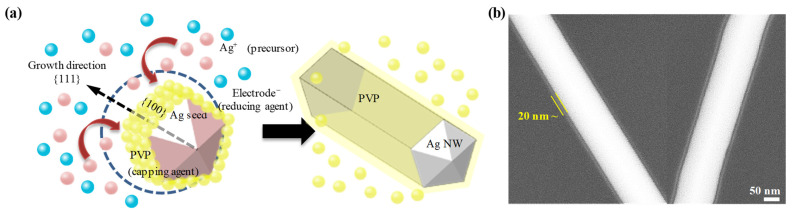
Synthesis of PVP-capped Ag NWs: (**a**) schematic of the Ag NW growth mechanism and (**b**) FE-SEM image of a PVP-capped Ag NW.

Figure 1 demonstrates the Ag NWs used in this work. The growth mechanism of Ag NWs by the polyol method is shown in Figure 1a. Ag NWs are grown from Ag seeds as Ag ions are reduced. It is noted that PVP serves as a capping agent to cover the (100) crystalline plane of the Ag seed and to grow in the (111) crystalline plane [50,51]. Generally, synthesized Ag NWs were rinsed with solvents to remove PVP before performing further processes. The resulting thickness of the PVP capping layer left on Ag NWs has been reported to be about 1 nm [52,53]. In this paper, however, we intentionally left a thicker PVP capping layer by using a mild washing scheme to increase the effect of selective calendering and to prevent oxidation that would result in an increase in electrical resistance. Figure 1b shows the Ag NW synthesized and washed in this study. A PVP layer of 20 nm thickness is clearly observed, the apparent difference can be seen between Ag NWs and PVP capped Ag NWs through the FE-SEM image [54]. Even though the thicker PVP hampers the percolation when Ag NWs are fabricated into a TCF, the calendering process successfully gives rise to a percolation path.

**Figure 2:**
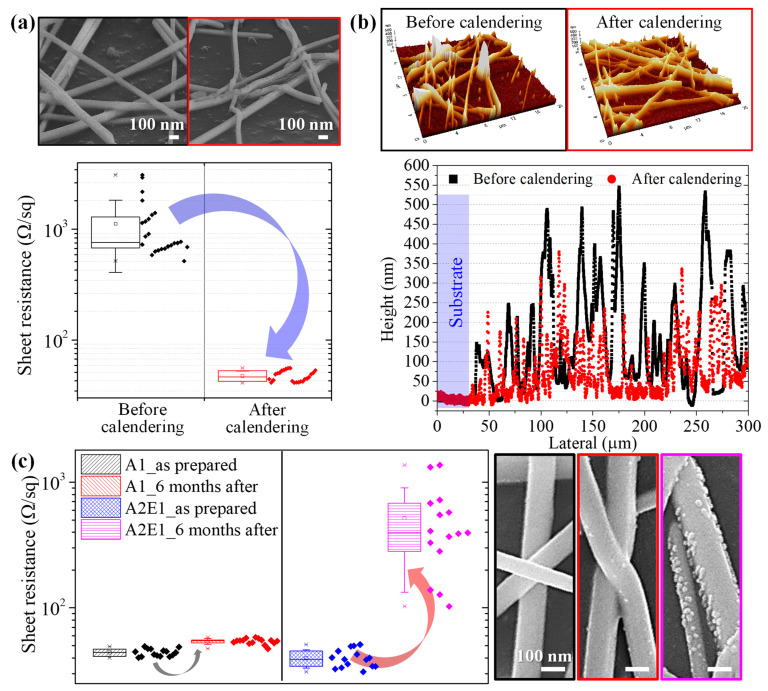
PVP-capped Ag NW TCFs calendered with flat roll: (**a**) FE-SEM images and sheet resistance change to demonstrate the effect of the calendering process, (**b**) AFM measurement of morphology. (**c**) FE-SEM images and sheet resistance change to show the effect of long-term oxidation when two different washing schemes (A1 and A2E1) were used.

The PVP-capped Ag NW TCFs were fabricated via R2R slot-die coating followed by a calendering process using a flat roll and the characterization result is displayed in Figure 2. The effect of the calendering process on the morphology and electrical properties of PVP-capped Ag NW TCFs is clearly demonstrated in Figure 2a. FE-SEM images depict a closer packing of the Ag NW random percolation network after calendering than as-coated. Furthermore, the average sheet resistance of PVP-capped Ag NW TCFs was significantly reduced from 1124 ± 700 to 47.4 ± 5.1 Ω/sq upon calendering. This drastic decrease in sheet resistance also confirms that the calendering process contributes to successful formation of a percolation path. The AFM measurement in Figure 2b shows a smoother surface after the calendering process than as-coated. While remarkable protruding Ag NW peaks are observed before calendering, they decreased significantly upon calendering, resulting in decreased surface roughness. Although silver itself has low reactivity among metals, their oxidation proceeds rapidly at nanoscale due to their high surface area [9]. This is one of the reasons that the PVP capping layer was left intentionally on the Ag NWs; to prevent oxidation. Various polymers and composites [55,56,57] have been generally used to passivate the surface. Figure 2c demonstrates the effect of washing, resulting in the long-term oxidation of PVP capped Ag NWs. To compare the effect of washing, two kinds of washing schemes were used after the synthesis of Ag NWs: one-time washing with acetone (denoted as A1) that was adopted in this work, and two-time washing with acetone followed by one-time washing with ethanol (denoted as A2E1) that has been adopted previously [9]. It is naturally expected that Ag NWs washed with A2E1 will have a thinner PVP capping layer [52] compared to that in Figure 1b. The oxidation stability was assessed alongside the sheet resistance immediately (as-prepared) and six months after production of calendered Ag NW TCF. The storage conditions were atmospheric pressure and ambient temperature. The washing scheme of A1 yielded an increased average sheet resistance (15 samples) of only 1.22 times, from 44.1 ± 3 to 54.1 ± 2.8 after six months storage. On the other hand, the A2E1 washing scheme returned an increased average sheet resistance (15 samples) of 13 times, from 39.8 ± 6.4 to 517.4 ± 371. It is noted that the lower sheet resistance was found in as-prepared Ag NWs with thinner PVP capping. However, a huge increase in the sheet resistance is observed in the A2E1 scheme, which presumably is due to oxidation of Ag NWs. The existence of nanoparticles on the surface of the oxidized Ag NWs has been proven through FE-SEM and EDS results [58,59,60] to be AgS nanoparticles generated by sulfur present in air. It can be seen that many more AgS particles are formed after six months of storage with the A2E1 scheme. Table 1 demonstrates the atomic percent of elements revealed from EDS and presents the oxygen stoichiometry of the compounds [61,62]. The element Ag comes solely from Ag NWs [63,64], while C, N, and O are associated with PVP based in the chemical structure of PVP ((C_6_H_9_NO)_n_) [65]. The amount of PVP left on the surface of Ag NWs is estimated by the atomic percent of nitrogen [66]. The as-prepared Ag NWs from two different washing schemes and Ag NWs after six months storage in an ambient environment were used for analysis. The as-prepared Ag NW-PVP composites have Ag:N ratios of 1:2.84 and 1:1.39 in the A1 and A2E1 scheme samples, respectively, which indicates that a thicker PVP capping layer is left in the A1 compared to the A2E1 sample. In addition, a higher atomic percent of O in the A2E1 scheme implies that oxidation is in progress. Oxidation induces a relatively high atomic percent ratio of oxygen with respect to the metal [67]. The Ag NW-PVP composite after six months storage has Ag:O ratios of 1:2.44 and 1:6.17 in the A1 and A2E1 schemes, respectively. This indicates that oxidation of silver occurs more severely in the A2E1 than in the A1 schemes, which ascribes to absence of a thick PVP capping layer. The existence of sulfur in the A2E1 scheme after six months storage also supports the formation of AgS nanoparticles that was evidence by FE-SEM. Therefore, the existence of a thick PVP capping layer protects Ag NWs from the oxidation that leads to high resistance [59]. Finally, the calendering of PVP-capped Ag NWs is an effective way to decrease junction resistance to attain a percolation path.

**Figure 3:**
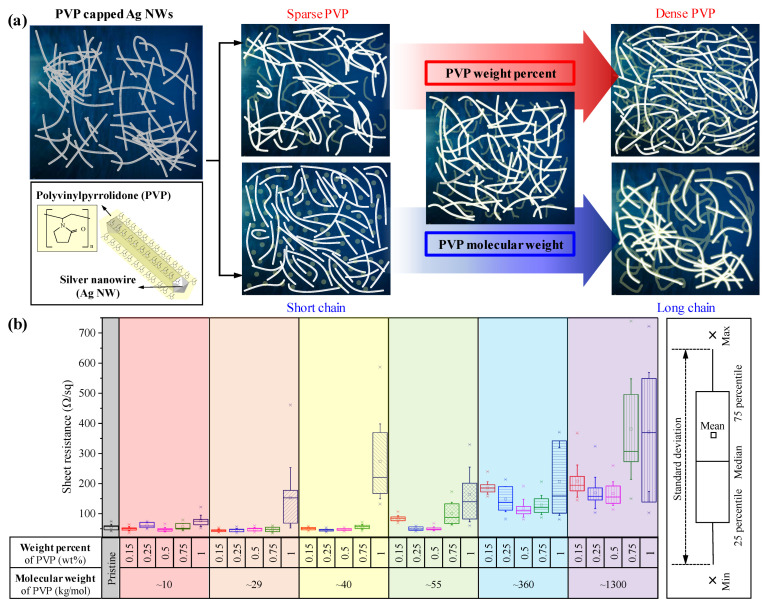
Dispersion of Ag NW-PVP composite solution with regard to PVP molecular weight and weight percent: (**a**) Schematic of Ag NW dispersion mechanism by adding PVP and comparison of (**b**) sheet resistance with box plot.

Since Ag NWs have an intrinsic aggregation problem, mixing with dispersants is mainly studied to solve this [68,69,70]. The dispersant prevents re-agglomeration of nanostructures by attaching to the surface, and at the same time acts as a factor that interferes with the properties of the raw material. We adopted PVP with various molecular weights as a dispersant for simplicity, since it is a surface regulating polymer that stabilizes the surface while acting as a dispersant to prevent agglomeration of the nanostructure [71,72,73]. On the one hand, the PVP might improve dispersion, which would result in uniform distribution of Ag NWs in coated film. On the other hand, excess PVP might be pinched among Ag NWs, which will decrease the conductivity of the coated film, which cannot be overcome by the calendering process. Important factors affecting the stability of dispersion are molecular weight, density, coexisting ions, and pH of the dispersant. We adopted different molecular weights and densities of PVP to investigate its effect on the electrical properties of Ag NW-PVP cTCFs, and the results are shown in Figure 3. Figure 3a depicts Ag NW-PVP cTCFs with respect to weight percent and molecular weight of PVP. As the concentration of PVP increases, the effect of dispersion is increased. However, at higher concentrations, the amount of PVP that can interact with the Ag NWs is limited, so residual PVP interrupts the percolation path among Ag NWs, which increases the sheet resistance of cTCF although the same pressure of calendering is applied. It can be checked in Figure 3b that the average sheet resistance and standard deviation rapidly increase at 0.75 wt% or more for all the different molecular weights. Specifically, the lowest sheet resistance was observed at 0.5 wt% of PVP. In addition, the effect of molecular weight was analyzed at a dispersant weight percent of 0.5 wt% representatively. As the molecular weight increased to 10, 29, 40, 55, 360, and 1300 kg/mol, the sheet resistance was measured at 47.7 ± 7, 48.5 ± 5.8, 47.4 ± 4.1, 50.0 ± 6, 118.4 ± 29.6, and 166.9 ± 39.3 Ω/sq, respectively. Although almost the same value of sheet resistance, within error, is observed at under ~55 kg/mol, the standard deviation becomes smaller. When the molecular weight is more than ~360 kg/mol, on the other hand, the sheet resistance increased drastically, which might be induced by the sparse packing of long PVP chains which hampers percolation among Ag NWs. In terms of quality control, a smaller standard deviation is preferred. Consequently, a PVP molecular weight of ~40 kg/mol and a concentration of 0.5 wt% represent optimized conditions to improve electrical conductivity by the calendering process.

### 3.2. Roll-to-Roll Continuous Patterning

**Figure 4:**
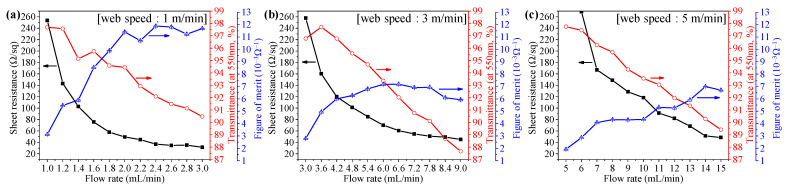
Sheet resistance and transmittance of Ag NW-PVP cTCFs fabricated with respect to flow rate in slot-die coating followed by full area calendering at web speeds of (**a**) 1, (**b**) 3, and (**c**) 5 m/min.

Figure 4 demonstrates the sheet resistance and transmittance of Ag NW-PVP cTCFs fabricated with respect to flow rate in slot-die coating followed by full area calendering at different web speeds. Since the calendering effect appears at a high web speed, the web speed was changed. The flow rate should be changed accordingly in slot-die module. To cover all areas of the web, a range of flow rates were tested at a certain web speed. The sheet resistance and transmittance decrease with an increase in flow rate at a certain speed. The figure of merit (FOM) is generally used to evaluate the performance of TCFs. The higher the FOM, the better the performance as a TCF. The FOM defined by Haacke was used, as shown in Equation (1) [74]:

FOM (Ω^−1^) = T^10^/R_sh_ (1)

where T is the transmittance and R_sh_ is the sheet resistance. As the flow rate increases at web speeds of 1, 3, and 5 m/min, the sheet resistance decreases in a different manner although the transmittance decreases in a similar manner. It seems that the addition of PVP has little influence on the transparency. However, sheet resistance decreased more at low web speeds compared to at high speed presumably due to a short calendering time. That is why the FOM at a web speed of 1 m/min is greater than that at 3 and 5 m/min. Furthermore, the FOM of Ag NW TCFs fabricated from thoroughly washed Ag NWs [9] was found to be a maximum of 5.81 mΩ^−1^. It confirms that addition of PVP into Ag NW solution increased dispersion of the Ag NWs, leading to better TCFs. This is the result of PVP thinly capped Ag NWs without PVP dispersant in the coating solution. After all, it ensures that a full online R2R process with a calendering effect according to various web speeds is stable, whereas it can be implemented by appropriately controlling the flow rate based on the desired sheet resistance and transmittance with regard to the processing speed.

**Figure 5:**
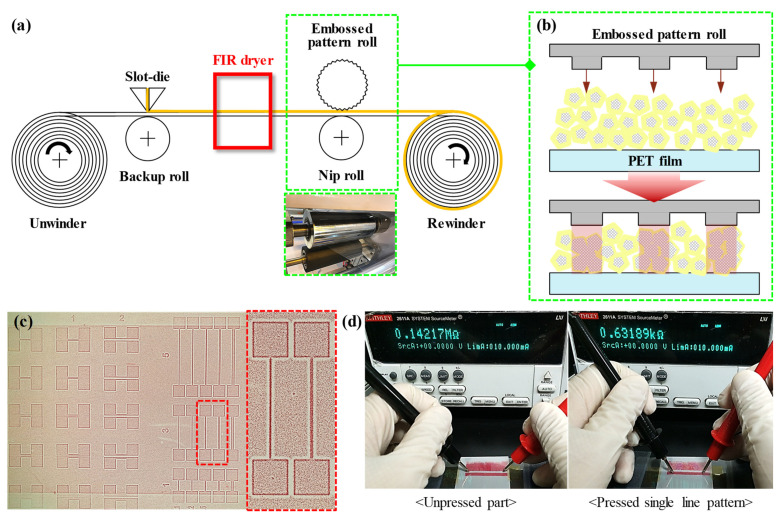
R2R-processed continuous patterning by selective calendering: (**a**) Schematic of concept for patterning process with R2R manufacturing and (**b**) selective calendering mechanism of PVP-Ag NW cTCF using an embossed pattern roll. (**c**) Pressure-sensitive paper pressed by an embossed pattern roll and (**d**) comparison of the resistance between the unpressed and pressed part in a single line pattern.

Figure 5a is a schematic diagram for patterning Ag NW-PVP cTCFs in a R2R process. Slot-die coated cTCF has a low electrical conductivity due to capped PVP without calendering. The patterning process is performed by pressing dried cTCF using an embossed pattern roll, in which only the pattern part is pressed, as shown in Figure 5b. Embossed pattern rolls are placed on the outfeeder. In order to show that pressure was placed on the pattern area, a pressure-sensitive paper was placed on the cTCF. The LLW model of Fujifilm PRESCALE was used because a high-resolution pressure-sensitive paper was required for the small width and spacing of line patterns. As the pressure is applied to the pressure-sensitive paper, it changes from white to red. In Figure 5c, it can be seen that the pattern area appears more red due to the high pressure. The nip roll supporting the embossed pattern roll is made of a rubber material, which causes the cTCF to bend and press areas other than the pattern. However, in the enlarged picture on the right of Figure 5c, the color of the pressure-sensitive paper was not changed close to 1 mm around the pattern, due to pressure not being applied because of the bending angle of the film. For this reason, the patterning was possible, since the pattern and other parts were disconnected, which means that no current flowed. The selective calendering process can be revealed by the difference in resistance between the unpressed and pressed parts of the single line pattern from Figure 5d.

**Figure 6:**
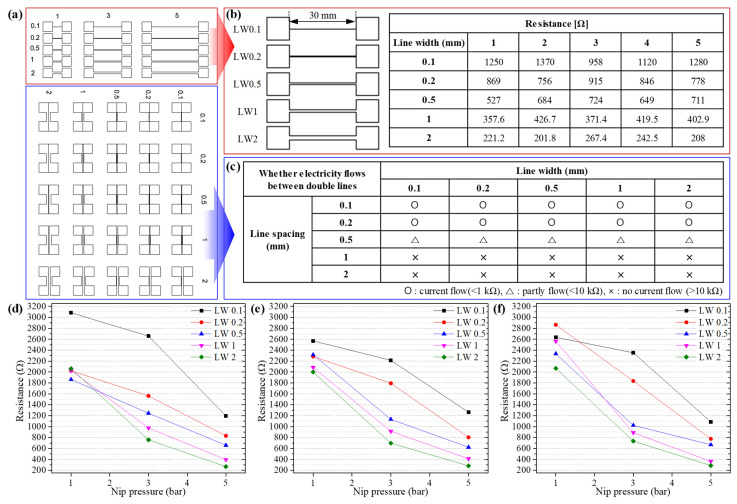
Experimental selective calendering for line and line spacing patterns: (**a**) design patterns for line and line spacing, (**b**) the resistance measurement of the line pattern with widths of 0.1, 0.2, 0.5, 1, and 2 mm, (**c**) measured resistance range from line spacing patterns with 0.1, 0.2, 0.5, 1, and 2 mm. The resistance of single line pattern with respect to the nip pressure at web speeds of (**d**) 1, (**e**) 3, and (**f**) 5 m/min.

Figure 6 shows an actual patterning test to determine the minimum line width and line spacing experimentally that can be achieved by selective calendering. The success of patterning was inspected with pressure-sensitive paper and by measuring the electrical resistance. There are two types of pattern sets as shown in Figure 6a. The upper panel shows designed line patterns with width of 100, 200, 500, 1000, and 2000 μm with a fixed length of 3 cm. The lower panel depicts designed pads set where the distance between the two pads is 100, 200, 500, 1000, and 2000 μm. The reason why we set the minimum width to 100 μm is the limitation of manufacturing. Using two sets of patterns, selective calendering was performed at a nip pressure of 5 bar followed by the measurement of resistance to confirm the success of patterning. Figure 6b,c demonstrates the resistance measurement results of line and line spacing patterns, respectively. As the line width increases from 100 to 2000 μm, the resistance decreases to 1195.6 ± 160.2, 832.8 ± 65.5, 659 ± 79.2, 395.6 ± 30.1, and 228.2 ± 26.9 Ω. The resistance is inversely proportional to line width. In the line spacing test of a double line pattern, three resistance ranges are defined: less than 1 kΩ is a short circuit, less than 10 kΩ is part flow, and more than 10 kΩ is insulation. More than 50 samples were tested for each condition for the purpose of determining the minimum line spacing at which the separated double line electrode is insulated. At a line spacing of 100 and 200 μm, the calendering effect occurred in a space between the two separated electrodes, resulting in a short circuit. On the other hand, the line spacing was insulated from more than 1000 μm, and the same tendency was observed regardless of line widths. The experimental minimum line width of a pattern that can be realized through selective calendering was determined to be 0.1 mm, and the line spacing was 1 mm. Since the nip roll used in this work is made of rubber, there is a large deformation during calendering, which set a limit in the minimum line width and line spacing. It can be complement the line width and spacing via the hardness of rubber material of the nip roll that is in contact with an embossed pattern roll. It is presumed that it can be improved by using a nip roll with high hardness rubber. Figure 6d–f is the resistance of line width with respect to nip pressure at the web speeds of 1, 3, and 5 m/min, respectively. As the nip pressure increases, the resistance of the line pattern generally tends to decrease and is distributed within ±10.3%, ±7.2%, and ±4.8%. There is little observed effect of web speed. Regardless of web speed, the higher the nip pressure indicates the more effective calendering.

### 3.3. Application as Functional Devices

**Figure 7:**
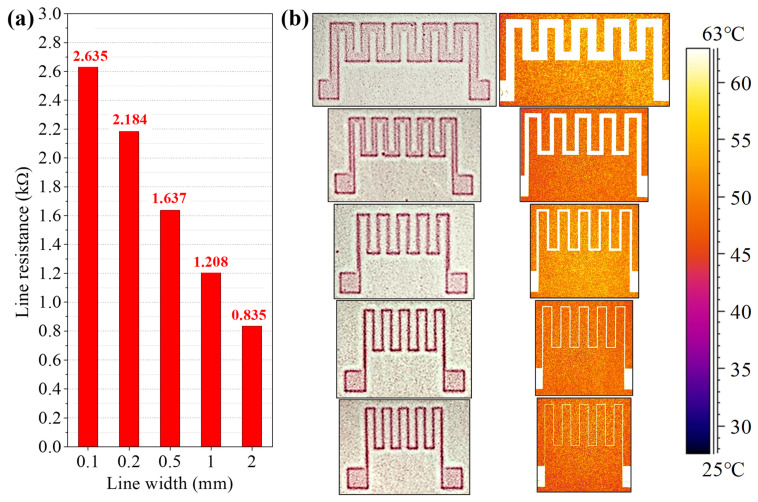
Zig-zag pattern for heating properties by using a continuous patterning process: (**a**) Resistance of pattern with regard to line widths of 0.1, 0.2, 0.5, 1, and 2 mm. (**b**) Picture of pressure-sensitive paper to check the selective calendering part (**left**), and far infrared camera thermal image for temperature distribution by Joule heating under an operating voltage of 5 V (**right**).

The flexible heater and touch sensing panel were fabricated to demonstrate the potential application of the selective calendering process in manufacturing of functional devices [75,76]. Figure 7 contains the result of flexible heater fabricated with a long, thin conductive strip in a zig-zag pattern. The line spacing was fixed at 1 mm, while the width was 0.1, 0.2, 0.5, 1, and 2 mm to compare heating performance. The resistance of each zig-zag patterns was 2635, 2184, 1637, 1208, and 835 Ω, respectively. It is natural that the resistance decreases as the line width increases. The left panel in Figure 7b shows the heater pattern pressed on pressure-sensitive paper, and the right panel depicts an infrared camera thermal image showing the temperature distribution of the heater when a voltage of 5 V is applied. The highest temperature was uniformly observed over the entire heater electrode patterns. The temperature rises to an average of 47.3 °C even in non-patterned areas excluding the pattern, which is mainly due to the thermal conduction from the pattern. In the heaters with line widths of 0.1, 0.2, 0.5, 1, and 2 mm, the heater temperature was measured to be 62.8 ± 0.82, 61.9 ± 0.66, 61.2 ± 0.61, 60.5 ± 0.59, and 58.5 ± 0.33 °C, respectively. As the resistance increases, the temperature range of the heater tends to decrease. The selective calendered Ag NW-PVP composite pattern can be utilized as a flexible heater.

**Figure 8:**
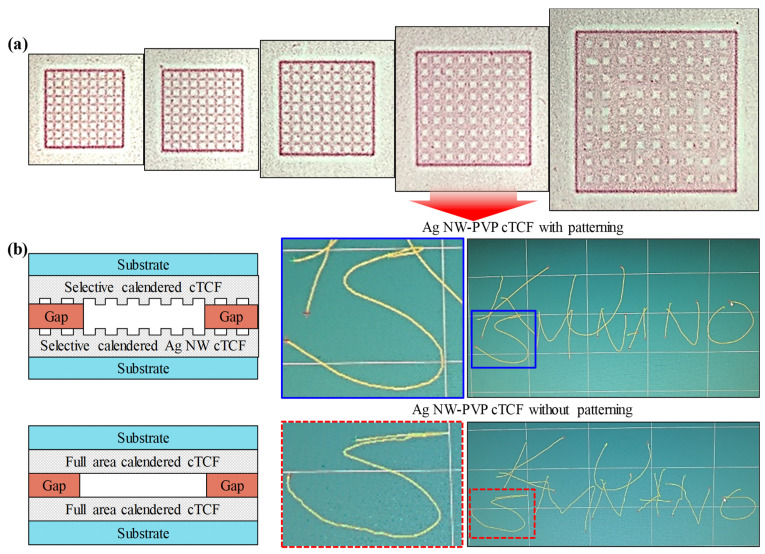
The resistive touch sensing panels fabricated by selective calendering of Ag NW-PVP cTCFs: (**a**) mesh pattern imaged on pressure-sensitive paper with various line widths, (**b**) cross-section structure and performance of touch sensing panel between selective calendered (upper) and full area calendered (bottom) Ag NW-PVP TCF with mesh pattern.

Figure 8 displays the demonstration of a resistive touch sensing panel. In order to apply selective calendering process effectively, a mesh pattern was exploited. We compared touch sensing panels manufactured by two methods: conventional full area calendering vs. selective calendering. It is noted that the substrate for the flexible touch panel is a flexible film such as PET, polyimide (PI), or polyether sulfone (PES), so the accuracy is relatively low due to the mechanical deformation. Figure 8a shows a picture of pressure-sensitive paper with a mesh pattern. The line spacing was fixed at 1 mm, and line widths of 0.1, 0.2, 0.5, 1, and 2 mm were chosen for selective calendering. As a result, the color of the pressure-sensitive paper was not uniform in about 20% of the sample at a line width of 2 mm. However, most of the samples were calendered uniformly when the line width was less than 1 mm. Furthermore, as the line width decreased, the resistance increased. Accordingly, a resistive touch panel was fabricated using a line width of 1 mm and line spacing of 1 mm mesh pattern as a representative. Figure 8b shows the cross-sectional view of the fabricated touch panel using all area calendering and selective calendering and the writing on the screen input from two kinds of touch sensing panels. Comparing the position accuracy of the touch panel, the letters are recognizable in both touch sensing panels. However, in the enlarged picture on the right, the full area calendered touch panel is relatively irregular compared to the selectively calendered touch panel. It proves that the mesh patterned Ag NW-PVP cTCF electrode improved the position accuracy of the resistive touch panel. It is highly probable that complex patterns are implemented by selective calendering, and the suggested patterning technology could find new applications in various electronic devices.

## 4. Conclusions

The continuous patterning of a Ag NW-PVP composite is developed based on R2R slot-die coating followed by the selective calendering performed by an embossed roll. The PVP plays a double role of a capping agent in the synthesis of Ag NWs and dispersant in the Ag NW-PVP coating solution. After it is calendered, the Ag NW-PVP composite film shows greatly decreased electrical resistance. The weight percent and molecular weight of PVP is optimized for calendered Ag NW-PVP composite film to have a low a sheet resistance as possible with small standard deviation. The optimized PVP properties are ~40 kg/mol of molecular weight and 0.5 wt% of weight percent for selective calendering. While the web speed has no significant effect on the decrease in resistance, a higher nip pressure produces a lower resistance. The patternable line width and spacing are found to be 0.1 mm and 1 mm, respectively. The selectively patterned Ag NW-PVP composite is successfully exploited as a functional layer in a flexible heater and a resistive touch sensing panel. A maximum temperature of 62.8 °C is observable in the calendered zig-zag line pattern while lower temperatures are maintained in non-calendered part. In the case of the resistive touch panel, the touch position accuracy is greatly increased when a meshed Ag NW-PVP composite pattern is used instead of all area calendered film. The R2R continuous patterning process by means of selective calendering can be utilized to manufacture fully functional flexible electronic devices.

## Figures and Tables

**Table 1:** The chemical elemental analysis data in accordance with as-prepared and oxidized A1 and A2E1 conditions.

Elements	Ag, at.w.%	C, at.w.%	O, at.w.%	N, at.w.%	S, at.w.%
A1_as prepared	4.32	74.0	9.31	12.30	-
A1_6 months after	4.13	76.05	10.07	9.76	-
A2E1_as prepared	8.79	61.08	17.90	12.24	-
A2E1_6 months after	1.89	86.21	11.67	-	0.22

## Data Availability

Not applicable.
